# Pancreatic pseudocyst with mediastinal extension: a rare cause of hiatal hernia

**DOI:** 10.1093/omcr/omab068

**Published:** 2021-09-13

**Authors:** Van Trung Hoang, Vichit Chansomphou, Tien Hoai Vo, Duc Thanh Hoang

**Affiliations:** 1 Department of Radiology, Thien Hanh Hospital, Buon Ma Thuot, Vietnam; 2 Department of Radiology, Savannakhet Medical-Diagnostic Center, Kaysone Phomvihane, Laos; 3 Department of Radiology, Tam Tri Nha Trang General Hospital, Nha Trang, Vietnam; 4 Division of Endocrinology, Department of Medicine, Walter Reed National Military Medical Center, Bethesda, MD, USA

**Keywords:** esophageal hiatus, hiatal hernia, mediastinal extension, pancreatic pseudocyst, paraesophageal hernia

## MANUSCRIPT

**Figure 1:**
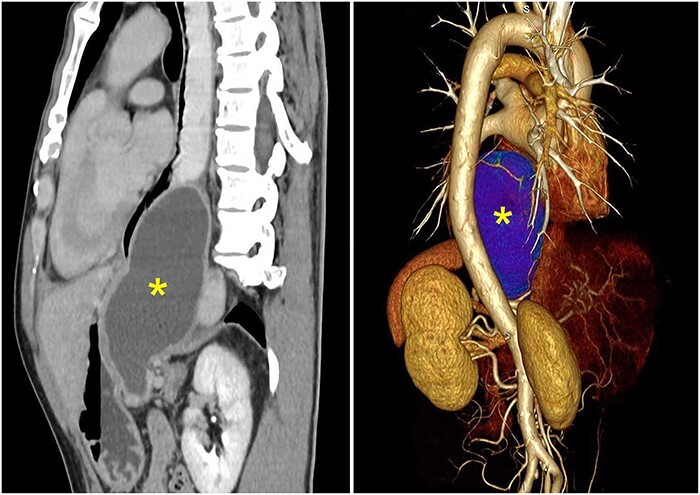
Sagittal and 3D-reconstruction CT images show a cystic structure extending from the pancreatic tail through the esophageal hiatus of the diaphragm into the mediastinum; this mass is located posteriorly to the esophagus and stomach, compressing the esophagus, and pushed the heart forward.

A 55-year-old man with a history of acute pancreatitis 6 months ago was admitted with symptoms of epigastric pain, chest tightness, dysphagia, weight loss and fatigue. Symptoms started a month ago and gradually worsened over the past week. Computed tomography (CT) images showed a cystic structure extending from the pancreatic tail through the esophageal hiatus of the diaphragm into the mediastinum. This cystic mass was 75 × 79 × 135 mm in size and had a rhombohedral shape with a light, irregular wall with thickness of about 3–5 mm. This cystic mass was located posteriorly to the esophagus and stomach, compressing the esophagus, and pushed the heart forward (Fig. 1). The final diagnosis was para-esophageal hernia of pancreatic pseudocyst based on the history, clinical presentation and imaging studies. The patient underwent a successful laparoscopic cystogastrostomy and paraesophageal hernia repair. At the 6-month follow-up, he showed improvement with a good appetite and weightgain.

Mediastinal pancreatic pseudocyst is a rare complication of acute or chronic pancreatitis and was first described in 1951 [1]. Mediastinal pancreatic pseudocysts are formed after rupture of the pancreatic duct into the retroperitoneal space and the pancreatic fluid moves into the mediastinum through the aortic or esophageal hiatus, resulting in clinical symptoms [2]. The treatment principle includes the management of pseudocyst, such as total excision with internal or external drainage via percutaneous, endoscopic, laparoscopic or open surgery (cystogastrostomy, cystojejunostomy, cystoduodenostomy or cystocholecystostomy) procedures [1–3]. Surgical approaches to paraesophageal hernia repair include laparoscopic, transabdominal, or transthoracic surgery [3]. The case of our patient revealed that nasopancreatic, transgastric or transesophageal endoscopic drainage was possible. However, the treatment was based on the experience and the equipment conditions of the hospital, so laparoscopic surgery had been applied to cystogastrostomy and hernia repair.

## FUNDING

No funding was obtained for this study.

## CONFLICT OF INTEREST

None declared.

## ETHICAL APPROVAL

No ethical approval required.

## CONSENT

Informed consent was obtained from the patient.

## GUARANTOR

Van Trung Hoang, Department of Radiology, Thien Hanh Hospital, Buon Ma Thuot, Vietnam.
